# Delay between Onset of Symptoms and Seeking Physician Intervention Increases Risk of Diabetic Foot Complications: Results of a Cross-Sectional Population-Based Survey

**DOI:** 10.1155/2016/1567405

**Published:** 2016-11-29

**Authors:** Norina A. Gavan, Ioan A. Veresiu, Etta J. Vinik, Aaron I. Vinik, Bogdan Florea, Cosmina I. Bondor

**Affiliations:** ^1^Society of Diabetic Neuropathy, Worwag Pharma GmbH&Co.KG, Romanian Representative Office, 11 Fagului Street, 400483 Cluj-Napoca, Romania; ^2^Department of Diabetes, Nutrition and Metabolic Diseases, Iuliu Hatieganu University of Medicine and Pharmacy Cluj-Napoca, 4-6 Clinicilor Street, 400006 Cluj-Napoca, Romania; ^3^Eastern Virginia Medical School, Strelitz Diabetes Center, 855 West Brambleton Avenue, Norfolk, VA 23510, USA; ^4^Research & Neuroendocrine Unit, Eastern Virginia Medical School, 855 West Brambleton Avenue, Norfolk, VA 23510, USA; ^5^IMOGEN Research Center, Iuliu Hatieganu University of Medicine and Pharmacy Cluj-Napoca, 8 Victor Babeș Street, 400012 Cluj-Napoca, Romania; ^6^Department of Medical Informatics and Biostatistics, Iuliu Hatieganu University of Medicine and Pharmacy Cluj-Napoca, 6 Pasteur Street, 400349 Cluj-Napoca, Romania

## Abstract

We present a post hoc analysis of 17,530 questionnaires collected as part of the 2012 screening for neuropathy using Norfolk Quality of Life tool in patients with diabetes in Romania, to assess the impact on foot complications of time between the onset of symptoms of diabetes/its complications and the physician visit. Odds ratios (ORs) for self-reporting neuropathy increased from 1.16 (95% CI: 1.07–1.25) in those who sought medical care in 1–6 months from symptoms of diabetes/its complications onset to 2.27 in those who sought medical care >2 years after symptoms onset. The ORs for having a history of foot ulcers were 1.43 (95% CI: 1.26–1.63) in those who sought medical care in 1–6 months and increased to 3.08 (95% CI: 2.59–3.66) in those who sought medical care after >2 years from symptoms of diabetes/its complications onset. The highest ORs for a history of gangrene (2.49 [95% CI: 1.90–3.26]) and amputations (2.18 [95% CI: 1.60–2.97]) were observed in those who sought medical care after >2 years following symptoms onset. In conclusion, we showed that waiting for >1 month after symptoms onset dramatically increases the risk of diabetic foot complications. These results show the need for accessible educational programs on diabetes and its chronic complications and the need to avoid delays in reporting.

## 1. Introduction

Diabetes represents a major worldwide epidemic that poses a great social, economic, and medical burden on both developed and underdeveloped countries [1]. An analysis of health examination surveys and epidemiological data involving 2.7 million participants performed in 2011 by Danaei et al. [2] showed that the number of people with diabetes doubled between 1980 and 2008, increasing from 153 million to 347 million. According to the International Diabetes Federation these figures further increased to 415 million in 2015 and are estimated to reach 642 million in 2040 [1]. Furthermore, it is estimated that 192.8 million people with diabetes have not been diagnosed and have an increased risk of developing complications, thus posing an additional burden on society [1]. The financial cost of diabetes is high. The estimated global health spending for treatment of diabetes and its complications for 2015 ranged between 673 and 1,197 billion USD [1]. An increase in the number of patients with diabetes has been also reported for Romania; the number of patients increased from 482,250 in 2005 to 803,489 in 2011 [3, 4]. The most recent epidemiological study performed in Romania between December 2012 and February 2014 showed that in adults 20 to 79 years of age the overall prevalence of diabetes adjusted for age and gender was 11.6% [5]. However, except for the analysis of the trends in the diabetes-related lower extremities [6], little information is available on the prevalence of its chronic complications or patient attitudes toward diabetes in Romania.

The Quality of Life in Patients with Diabetic Neuropathy in Romania was a cross-sectional, noninterventional, multicenter survey performed with the involvement of 181 healthcare professionals and aimed to capture undiagnosed neuropathy in patients with self-reported diabetes in Romania by using the Norfolk Quality of Life (Norfolk-QOL-DN) questionnaire as a screening tool [7]. This survey revealed a high prevalence of undisclosed neuropathy (50%) [7], as well as a high prevalence of foot ulcers (14.85%) and amputations (3.60%) in this population [8].

Here we present a post hoc analysis of data collected in this survey aiming to assess diabetes chronic complications (i.e., neuropathy, foot ulcers, gangrene, and amputations) as a function of time between the onset of symptoms of diabetes or its complications and the physician visit for those symptoms. At this time, no studies in Romania have assessed this association in patients with neuropathic symptoms. These results may help to fill the knowledge gap on patients' health beliefs and provide invaluable support for developing future educational programs aimed at preventing diabetes complications.

## 2. Materials and Methods

The methodology of this cross-sectional survey performed between January and December 2012 was previously described elsewhere [7]. Briefly, self-administered questionnaires were distributed by physician specialists in diabetes, neurologists, general practitioners, and nurses from all regions in Romania to their patients with diabetes. Data were collected using the Romanian version of the Norfolk QOL-DN questionnaire [7]. Of the 25,000 questionnaires distributed, 23,543 were returned and fully completed ones were entered in the database.

The questionnaire used comprises 35 scored items reflecting patients' health perception and used to calculate the total Norfolk QOL-DN score and 5 subdomain scores for symptoms, activities of daily living (ADLs), autonomic neuropathy, physical functioning/large fiber neuropathy, and small fiber neuropathy [5]. Additionally, the questionnaire has items which are not scored and were used to collect demographic (age, gender) and medical history information [7].

The analysis presented here contains the responses to the following medical history questions. “Do you have diabetes?” “Do you have neuropathy?” “Have you ever had an ulcer on your feet?” “Have you ever had gangrene?” “Have you had any toes or fingers amputated?” “How soon after the onset of the first symptoms of diabetes/its complications did you make an appointment for a physician visit and see the physician?” The patients were asked to respond with “Yes” or “No” to all these questions, except for the last one in which the patients were asked to choose among the 6 possible responses:

- less than 1 month,
- between 1 and 6 months,
- between 6 and 12 months,
- between 1 and 2 years,
- over 2 years,
- I do not know/I do not remember.

Before completion of questionnaires, the patients were informed that their personal data would be collected as part of this survey and consented for their data to be analyzed. The survey was approved by The National Supervisory Authority for Personal Data Processing under the number 0006753.22-03-2012.

### 2.1. Main Objective

The main objective of the analysis presented here was to assess the association between the presence of self-reported neuropathy, foot ulcers, gangrene and amputations and the time interval between the onset of symptoms of diabetes or its complications and the physician visit for those symptoms.

### 2.2. Statistical Analysis

For the analysis presented here we included only those questionnaires which provided “Yes” as an answer to the question “do you have diabetes?” Of these, only those with an answer other than “I do not know/I do not remember” to the question “how soon after the onset of the first symptoms of diabetes/its complications did you make an appointment for a physician visit and see the physician?” were included in the analysis.

Qualitative variables were summarized with frequency tables. Descriptive statistics (mean, standard deviation, and standard error, minimum, and maximum) were calculated for continuous variables. The age and total Norfolk QOL-DN and subdomain scores were compared between categories of responses to the questions “how soon after the onset of the first symptoms of diabetes/its complications did you make an appointment for a physician visit and see the physician?” by student *t*-test and Kruskal Wallis test. Gender, frequency of self-reported neuropathy, foot ulcers, gangrene, and amputations were compared by Chi-square test.

The association of the time interval between patient symptoms onset and physician visit for those symptoms with self-declaring the presence of neuropathy and the probability a history of foot ulcers, gangrene, or amputations was tested by logistic regression while controlling for age and sex. Additionally, the models with the history of foot ulcers, gangrene, or amputations as dependent variables were adjusted for the presence of neuropathy.

Two-way bifactorial ANOVA with age and gender as covariates was used to test the influence of time interval between symptoms onset and physician visit on the total Norfolk QOL-DN and the presence of self-reported neuropathy. The age and sex were used as covariates due to significant differences between groups in terms of age observed in the current analysis and the differences in the total Norfolk QOL-DN score in men and women previously observed in our sample [7]. The estimated marginal means of the Norfolk QOL-DN total score adjusted for age and sex calculated with this model were further compared with the cut-off previously used as suggestive for the presence of diabetic neuropathy [7].

All statistical analyses were performed using IBM SPSS Statistics for Windows, Version 15.0 (Armonk, NY: IBM Corp). A *p* value < 0.05 was considered statistically significant.

## 3. Results and Discussions

### 3.1. Results

**Figure 1:**
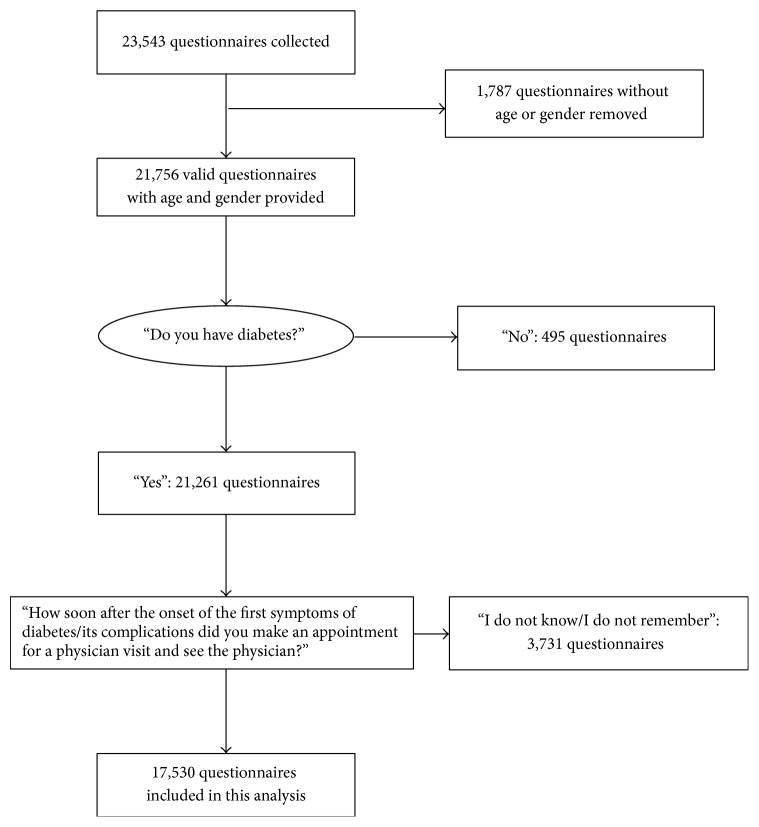
Participant flow diagram.

As previously described [7], 23,543 completed questionnaires were collected. Of these, 21,756 had age and gender provided and were considered valid. After removing those with a missing answer or “No” as an answer to the question “do you have diabetes?” (495 questionnaires; 2.3% of the valid questionnaires) and those with a missing answer or “I do not know/I do not remember” as an answer to the question “how soon after the onset of the first symptoms of diabetes/its complications did you make an appointment for a physician visit and see the physician?” (3,731 questionnaires; 17.1%), 17,530 questionnaires were included in the analysis presented here (Figure 1).

**Figure 2:**
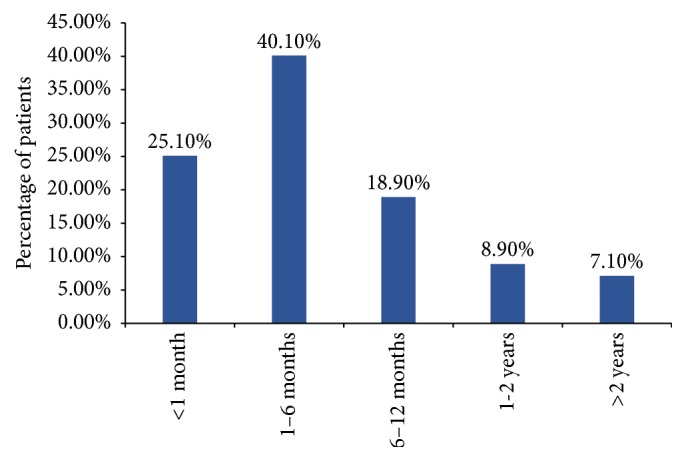
The time interval between symptoms of diabetes/its complication onset and physician visit for those symptoms.

The majority of patients reported having sought medical care within 6 months after the onset of symptoms of diabetes/its complications: 4,401 of 17,530 (25.1%) reported having sought medical care within 1 month after the symptoms of diabetes/its complications onset and 7,023 of 17,530 (40.1%) between 1 and 6 months after the onset of symptoms. However, 1,558 of the 17,530 included in the analysis (8.9%) and 1,239 of the 17,530 included in the analysis (7.1%) reported having sought medical care only after 1 to 2 years and more than 2 years after the symptoms of diabetes/its complications onset, respectively (Figure 2).

No difference was observed in the time interval between symptoms of diabetes/its complications onset and physician visit in terms of gender (Table 1). Persons who sought medical care after more than 1 year following symptom onset were significantly older than those who sought medical care less than 1 month from symptom onset (*p* < 0.001).

The percentage of patients with a history of foot ulcers, gangrene, and amputations increased with the time interval between the symptoms of diabetes/its complications onset and the physician visit for those symptoms. The percentage of patients with a history of foot ulcers increased from 8.8% in the groups who sought medical care <1 month from symptom onset to 12.4% in those who sought medical care between 1 to 6 months, 16.0% in those who sought medical care between 6 and 12 months from symptom onset, 20% in those who sought medical care between 1 and 2 years, and 27.0% in those sought medical care after 2 years. For gangrene and amputations, the percentage increased from 3.1% and 2.5%, respectively, in those who sought medical care within 1 month to 8.6% and 6.1%, respectively, in those who sought medical care after more than 2 years. A similar trend was also observed for self-reported neuropathy on the prevalence of foot complications (Table 1).

**Figure 3:**
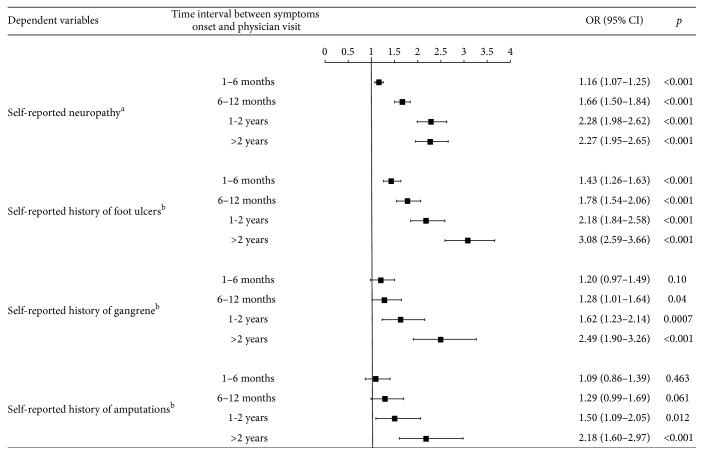
Forest plot for the probability of declaring a history of neuropathy, foot ulcers, gangrene, and amputations according to the time interval between onset of symptoms of diabetes/complications and physician visit. Category <1 month was considered as reference in the model. ^a^Regression model adjusted for age and gender. ^b^Regression model adjusted for the presence of self-reported neuropathy, age, and gender. OR: odds ratio; CI: confidence interval.

The odds of self-reporting the presence of neuropathy were significantly higher in those who sought medical care later than 1 month from symptoms of diabetes/its complications onset as compared to those who sought medical care within 1 month from symptoms onset. Odds ratios (ORs) for reporting neuropathy increased from 1.16 (95% confidence interval [CI]: 1.07–1.25) in those who sought medical care between 1 and 6 months from symptoms of diabetes/its complications onset to 2.28 and 2.27 in those who sought medical care in 1 to 2 years or in more than 2 years after symptoms onset. The ORs for having a history of foot ulcers were also significantly higher in those who sought medical care after 1 month from symptoms of diabetes/its complications onset: 1.43 (95% CI: 1.26–1.63) in those who sought medical care in 1 to 6 months, 1.78 (95% CI: 1.54–2.06) in those who sought medical care in 6 to 12 months, 2.18 (95% CI: 1.84–2.58) in those who sought medical care in 1 to 2 years, and 3.08 (95% CI: 2.59–3.66) in those who sought medical care after more than 2 years from symptom onset. The ORs for having a history of gangrene were significantly higher as compared to the reference category (those who sought medical care in less than 1 month from symptom of diabetes/its complications onset) only in patients who sought medical care more than 6 months from symptom onset. The ORs for having a history of amputations were significantly higher as compared to the reference category only in patients who sought medical care more than 1 year from symptom of diabetes/its complications onset. The highest ORs for a history of gangrene and amputations were observed in those who sought medical care after more than 2 years following symptom onset: 2.49 (95% CI: 1.90–3.26) for gangrene and 2.18 (95% CI: 1.60–2.97) for amputations (Figure 3).

**Figure 4:**
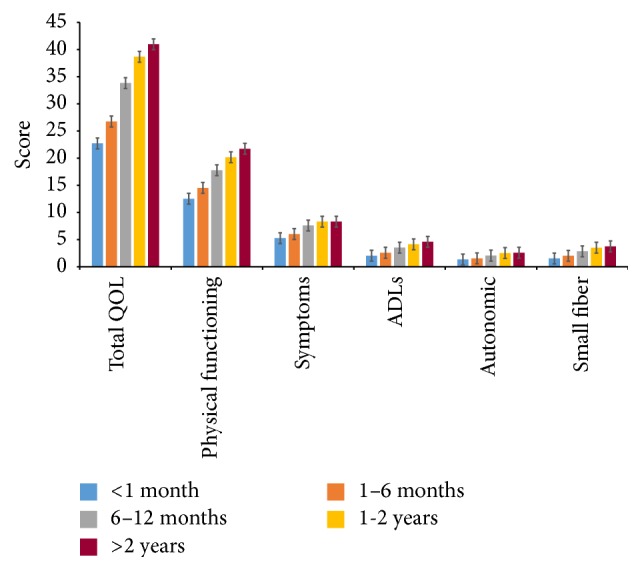
Norfolk QOL-DN total and subscale scores in Romanian patients with self-reported diabetes mellitus according to the time interval between onset of symptoms of diabetes/complications and physician visit for those symptoms. QOL: quality of life; ADLs: activities of daily living. *p* < 0.001 for trend for Norfolk QOL-DN total and subscale scores.

The mean scores for total Norfolk QOL-DN and all subdomain scores increased significantly and in parallel with the time interval between the symptoms onset and the physician visit for those symptoms (Figure 4, *p* < 0.001 for all). For all these comparisons, the lower scores, which represent better QOL, were observed in those who sought medical care within 1 month from symptoms of diabetes/its complications onset: 22.72 for total Norfolk QOL-DN score; 12.53 for physical functioning/large fiber neuropathy; 5.27 for symptoms; 2.05 for ADLs; 1.34 for autonomic neuropathy; and 1.53 for small fiber subdomain. The maximum score, indicating poorer QOL, was observed in those who sought medical care more than 2 years after the symptom onset: 40.96 for Norfolk QOL-DN score; 21.72 for physical functioning/large fiber neuropathy; 8.31 for symptoms; 4.59 for ADLs; 2.59 for autonomic neuropathy; and 3.75 for small fiber subdomain.

**Figure 5:**
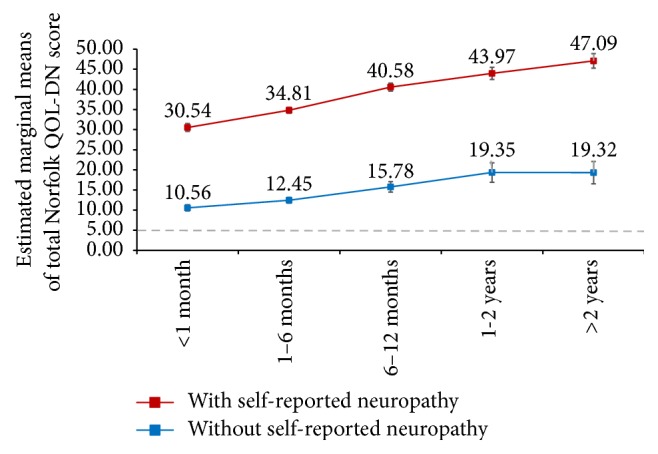
Estimated marginal means of Norfolk QOL-DN total score in Romanian patients with self-reported diabetes mellitus with and without neuropathy according to the time interval between onset of symptoms of diabetes/complications and physician visit for those symptoms after controlling for age and gender. Error bars represent 95% confidence interval of the estimated marginal means of the total Norfolk-QOL score. Dashed line represents the total Norfolk-QOL score cut-off value suggestive for the presence of neuropathy [7].

The ANOVA analysis confirmed that the time between symptoms of diabetes/its complications onset and physician visit for those symptoms had a significant impact on the total Norfolk QOL-DN score and that this association was independent of age and gender. A significant interaction was also observed in this model between the Norfolk QOL-DN and self-reported neuropathy (Figure 5). The estimated marginal means of Norfolk QOL-DN, adjusted for age and sex, in those with self-reported neuropathy were 30.54 for those who sought medical care within 1 month after symptoms of diabetes/its complications onset and increased to 47.09 in those who sought medical care after more than 2 years from symptoms onset. A similar trend was observed in patients without self-reported neuropathy. The estimated marginal means of Norfolk QOL-DN, adjusted for age and sex, increased in parallel with time interval between symptoms of diabetes/its complications onset and a physician visit (ranging from 10.56 in those who sought medical care in less than 1 month after symptoms onset and 19.35 in those who sought medical care between 1 and 2 years after the symptoms onset). All estimated marginal means of Norfolk QOL-DN were higher than the cut-off previously used suggestive for the presence of neuropathy [7].

### 3.2. Discussion

The results of this analysis of the data collected in the screening for neuropathy survey using the QOL-DN Norfolk tool in patients with diabetes in Romania showed that the majority of patients sought medical care after only 1 month from the onset of symptoms of diabetes or its complications, while 33% waited for more than 6 months before addressing a physician and 16% sought medical care only after 1 year.

We found a significant association between the time between the occurrence of symptoms of diabetes/its complications and the consult with a physician and the occurrence of self-reported neuropathy, foot ulcers, gangrene, and amputations, confirming previously observed results. The odds of self-reported neuropathy and foot ulcers were significantly higher in those who delayed seeking medical care for more than 1 month after the onset of symptoms of diabetes or its complications as compared to those presenting within 1 month from symptoms onset and increased in parallel with the time between the symptoms onset and the physician visit. Notably, the risk of more serious complications, such as gangrene and amputations, became significantly higher in those who sought medical care after 1 year from symptoms onset and more than doubled in those who sought medical care after 2 years from symptoms onset.

Previously we found a high prevalence (52.5%) of undisclosed diabetic neuropathy in this population [7]. The results of the analysis presented here show that the high prevalence of the undisclosed neuropathy is partially attributable to patients who do not report their symptoms to the healthcare providers or report them late after the symptoms onset, thus showing the limited efficacy of existing educational programs in diabetes in Romania. This is in line with results of previous studies and surveys which showed that diabetes is often perceived by patients as a nonserious disease until complications occur [9–11]. According to the Health Belief Model, a person will probably take action toward a disease if he/she perceives themselves to be at risk for the disease, is aware of the severity of a condition and of the benefits of certain actions, and is given cues for actions [12]. Additionally, identifying barriers and practical ways to overcome them is also important [13]. Previous studies in patients with diabetes showed that a patient-centered multidisciplinary educational approach adapted to their level of understanding is effective in increasing the compliance and adherence to diabetes management and preventive care of chronic complications [14–17] and has a positive impact on reducing the diabetes-associated complications and costs [18–24]. Here we show that the answer to a single question on a positive response to the development of symptoms of diabetes/its complications should alert the individual to seek care forthwith. Our results clearly demonstrate that delaying more than one month can have a disastrous outcome on the foot complications of diabetes and education programs should impart this simple message.

The type of symptoms experienced by patients may be another cause of delay between onset of symptoms and seeking physician intervention. In a previous analysis of this survey (Gavan et al., Symptom Characteristics That Alert Patients to the Diagnosis of Diabetic Neuropathy, 2015, submitted), we identified a group of patients who self-reported neuropathy without being diagnosed by a physician. This group reported more frequently symptoms in hands and arms than the group with a previous diagnosis of neuropathy who reported more frequently symptoms in legs and feet. A higher likelihood of the patients to self-report neuropathy although they had not been told they had this diagnosis was also associated with autonomic neuropathy, large fiber neuropathy, and ADLs subdomain scores. The presence of symptoms in feet and legs usually alerts patient to their neuropathy and probably these patients sought for medical care soon after the symptoms onset. The group of patients with symptoms in hands and arms, autonomic neuropathy, or symptoms attributed to small fiber neuropathy may have failed to recognize the defined symptoms or, if recognized, did not report them to their physician.

The mean total Norfolk QOL-DN score in those who sought medical care within 1 month from symptoms of diabetes/its complications onset was similar to those previously reported in patients with neuropathy without symptoms (22.72) and the mean total Norfolk QOL-DN score in those who sought medical care after more than 2 years after symptoms onset (40.96) was similar to the ones of a group with symptomatic neuropathy in a German population [25]. These results confirmed once gain the deleterious impact that delays in diagnosis and treatment have on the progression of diabetic neuropathy.

Another cause for the delayed appointment to a physician after the symptoms of diabetes/its complications onset may be the limited access to foot examination in the diabetic population [7]. The foot examination in primary care is limited in both Romania and elsewhere [26–28]. Although as per Romanian medical system organization each patient with diabetes should be seen by their primary care physician or a physician specialist in diabetes every 3 months, we have reasons to believe that the majority of them do not have a foot examination performed at least once a year. This may also have a negative impact on the patient's perception on the importance of seeking medical care when symptoms of diabetes or its complications occur. A study performed as part of the Quality of Care and Outcomes in Type 2 Diabetes project aiming to assess the physician's attitude towards foot care education and foot exam in patients with type 2 diabetes showed that foot examination was performed less frequently by general practitioners as compared to physician specialists in diabetes and more frequently in those with foot complications (not diabetic neuropathy) [27]. Additionally, it was shown that patients who had a foot exam were more likely to check their feet regularly [27]. Educational programs targeting physicians have been shown to increase the performance of screening activities [26] and thus may have a positive impact on reducing the time between the onset of symptoms of diabetes or its complications and medical care.

Notwithstanding that our survey has strengths derived from the large number of patients included and which makes its results representative for Romanian patients with diabetes, it also has limitations. Although it would have provided more detailed information, we did not ask for separate answers for the time between the diabetes symptoms onset and the physician visit for those symptoms and between the time of symptoms of complications onset and the physician visit for those symptoms. We have not asked either specific question to assess which diabetes complication the patients referred to when answering the following question: “how soon after the onset of the first symptoms of diabetes/its complications did you make an appointment for a physician visit and see the physician?” We chose this approach to assess patients' beliefs, education, and knowledge and the overall attitude toward the health status and the primary and secondary prevention of diabetes and its complications. Therefore, we do not know if the patient answers referred to the time between the onset of symptoms of diabetes alone or its complications and the moment of seeking medical care for these symptoms. As we previously mentioned the Norfolk QOL-DN questionnaire was specifically designed for the evaluation of the impact of diabetic neuropathy on QOL and showed good sensitivity, specificity, and positive and negative predictive values for the severity of neuropathy [25, 29, 30]. In addition, this tool has been used in Romania to screen for diabetic neuropathy [7]. It has also been used to monitor QOL in patients with amyloid neuropathy [31]. Here we used an additional question to define the onset of symptoms of diabetes/complications which did not correspond with the items of the Norfolk QOL-DN. It remains to be ascertained more specifically what the content of the question on diabetes and its complications would be the most useful in proposing an education program to reduce the latency of patients' decision to visit a physician. However, we based our analysis on the questions asked of patients and their self-reported responses and not on reviewing medical charts or objective measures of neuropathy and its complications recall bias cannot be excluded and more specifically must depend on the nature of the question itself.

## 4. Conclusions

In conclusion, we showed that waiting for more than 1 month after symptom onset of diabetes/neuropathy dramatically increases the risk of neuropathy, foot ulcers, gangrene, and amputations. Seventy-five percent of the patients who completed the questionnaires waited for more than 1 month after the symptom onset to seek medical attention, and 16% sought medical attention after 1 year following the symptom onset. These results are alarming and support the need to implement easily accessible educational programs on diabetes and its chronic complications.

## Figures and Tables

**Table 1:** Demographic characteristics and history of self-reported neuropathy, foot ulcers, gangrene, and amputations according to the time interval between symptoms of diabetes/its complication onset and physician visit for those symptoms.

	*N*′	Time between symptom onset and physician visit for those symptoms					Total *N* = 17,530	*p*
		<1 month *N* = 4,401	1–6 months *N* = 7,023	6–12 months *N* = 3,309	1-2 years *N* = 1,558	>2 years *N* = 1,239		
Women, *n* (%)	17,490	2,274 (51.8)	3,700 (52.8)	1,789 (54.2)	833 (53.6)	678 (54.8)	9,274 (53.0)	0.18
Age, years Mean ± SD	17,530	58.7 ± 12.3	60.3 ± 11.2	61.6 ± 10.7	62.2 ± 10.5	62.1 ± 10.1	60.5 ± 10.1	<0.001
Diabetes with self-reported neuropathy, *n* (%)	16,928	2,568 (60.7)	4,370 (64.7)	2,360 (72.9)	1,202 (78.9)	934 (78.8)	11,434 (67.5)	<0.001
Diabetes with history of foot ulcers, *n* (%)	17,242	382 (8.8)	853 (12.4)	526 (16.0)	308 (20.0)	328 (27.0)	2397 (13.9)	<0.001
Diabetes with history of gangrene, *n* (%)	17,240	135 (3.1)	266 (3.9)	148 (4.5)	93 (6.1)	105 (8.6)	747 (4.3)	<0.001
Diabetes with history of amputations, *n* (%)	1,7251	110 (2.5)	199 (2.9)	120 (3.7)	70 (4.6)	74 (6.1)	573 (3.3)	<0.001

*N* = number of patients in given category; *N*′ = number of patients with available responses to a given question; *n* (%) = number (percentage); SD = standard deviation.
